# Lactobacillus salivarius Promotion of Intestinal Stem Cell Activity in Hens Is Associated with Succinate-Induced Mitochondrial Energy Metabolism

**DOI:** 10.1128/msystems.00903-22

**Published:** 2022-11-22

**Authors:** Zhou Zhou, Lingzi Yu, Jiajia Cao, Jiaming Yu, Zhibo Lin, Yi Hong, Sibo Jiang, Cong Chen, Yuling Mi, Caiqiao Zhang, Jian Li

**Affiliations:** a Department of Veterinary Medicine, College of Animal Sciences, Zhejiang University, Hangzhou, People’s Republic of China; b Zhejiang Provincial Key Laboratory of Preventive Veterinary Medicine, College of Animal Sciences, Zhejiang University, Hangzhou, People’s Republic of China; c Yanping Bureau of Agriculture and Rural Affairs, Nanping, People’s Republic of China; University of Maine

**Keywords:** *Lactobacillus*, intestinal stem cell, succinate, mitochondria, energy metabolism

## Abstract

Currently, the regulation of *Lactobacillus* on intestinal stem cells (ISCs) attracts broad attention, but their active ingredients and the underlying mechanism are worthy of further study. Previously, host intestinal commensal bacteria were verified to drive the differentiation of ISCs. In this study, the strong bacteriostatic activity of Lactobacillus salivarius and *Lactobacillus agilis* were illustrated, and the components (supernatant, precipitation) of *L. salivarius* or *L. agilis* were further demonstrated to decrease the differentiation of ISCs *in vivo*. Interestingly, antibiotics feeding decreased ISCs differentiation *in vivo* as well. However, the administration of *L. salivarius* supernatant following antibiotics feeding was shown to promote ISCs differentiation dramatically when compared with the antibiotics feeding group, indicating that some active ingredients existed in its supernatant to promote ISCs activity. Strikingly, *in vitro*, the treatment of *L. salivarius* supernatant was further confirmed to promote the intestinal organoids’ size, budding, and LGR5 expression. Next, the metabolomics analysis of *Lactobacilli’* supernatants suggested that succinate might be a crucial metabolite to promote ISCs activity. Further, the succinate treatment *in vitro* (1000 μM) and *in vivo* (50 mM) was confirmed to enhance the expression of LGR5 and PCNA. *SLC13A3* (a sodium/dicarboxylate cotransporter) was detected in the intestinal organoids and demonstrated to transport succinate into ISCs, as confirmed by the contact of FITC-succinate with ISCs nucleus. Subsequently, high mitochondrial membrane potential and reactive oxygen species levels appeared in the intestinal organoids upon succinate treatment. Collectively, the promotion of *L. salivarius* on ISCs activity is associated with succinate-induced mitochondrial energy metabolism.

**IMPORTANCE** In our previous study, Lactobacillus salivarius and *Lactobacillus agilis* were demonstrated to regulate intestinal stem cell activity in hens, but their active ingredients and the underlying mechanism remain unclear. In this study, *L. salivarius* supernatant was shown to directly promote intestinal stem cell activity. Furthermore, the succinate (a critical metabolite of *L. salivarius*) was screened out to promote intestinal stem cell activity. Moreover, the succinate was confirmed to enter intestinal stem cells and induce high mitochondrial energy metabolism, finally promoting intestinal stem cell activity. These findings will advance uncovering the mechanism by which *Lactobacillus* regulate intestinal stem cell activity in chickens.

## INTRODUCTION

The rapid mucosal renewal relies on the proliferation and differentiation of the intestinal stem cell (ISCs). At the bottom of the intestinal crypt, there exist two types of ISCs. The active ISCs (aISCs), interspersed between Paneth cells, were marked by *Lgr5*, and the reserve ISCs (rISCs), located at the “+4” position directly above the Paneth cells, were marked by *Bmi1*, *mTert*, *Hopx*, and *Lrig1*, etc., (1, 2). The aISCs maintained continuous self-renewal and contributed to daily mucosal renewal, whereas rISCs quiescence under physiological state and would transform to aISCs after injury (3–6). The activity of ISCs was regulated by niche, which is mainly composed of Paneth cells that provide lactate, Wnt3, EGF, and Notch ligands (Dll1 and Dll4) (7). Rodríguez-Colman et al. (8) corroborated that lactate produced by Paneth cells could diffuse into the neighboring LGR5^+^ ISCs and further convert into Pyruvate to fuel mitochondrial oxidative phosphorylation (OXPHOS), leading to increased reactive oxygen species (ROS) signaling and finally promoting the differentiation of aISCs. Furthermore, several studies showed that probiotics could alter the ISCs niche. For instance, oral administration of probiotics was demonstrated to increase the activity of Paneth cells (9). *L. plantarum-*derived lactate was proved to stimulate mice’s ISCs proliferation through increasing mitochondrial function-related respiration in the crypts and activating Wnt3/β-catenin signals in ISCs (10). In the poultry industry, it is indefinite which *Lactobacillus* can target regulating ISCs and further enhance mucosal function. Addressing this issue will provide an innovative way to improve laying performance in a nonantibiotic manner.

*Lactobacillus* has been widely used in the poultry industry to ameliorate egg-laying performance and egg quality. In our previous studies, predominant *Lactobacilli* (*L. salivarius* and *L. agilis*), which were screened from native hens and fed in HyLine hens, were demonstrated to improve egg-laying rate, egg weight, and albumen’s amino acid levels (11, 12). Similarly, *L. salivarius* CML352 (isolated from Chinese native chicken) and *L. agilis* were proved to improve egg quality or increase weight gains in chicken (13, 14), feeding of *L. plantarum* or cofeeding of *L. pentosus* ITA23 and L. acidophilus ITA44 were proved to strengthen intestinal mucosal absorption (15, 16). It is well known that the mucosal absorption function relies on rapid mucosal renewal, which depends on the activity of ISCs. Recently, ROS have emerged as critical intracellular messengers that drove the differentiation of ISCs (17). Our previous study found that cofeeding of *L. salivarius* and *L. agilis* could promote the crypt’s local ROS levels and regulate ISCs activity in hens (12). In contrast, LPS (lipopolysaccharide)-induced intestinal inflammation resulted in mitochondrial swelling in Paneth cells at the early stage of inflammation, consequently reducing energy metabolism in the niche and thus descending the activity of ISCs (18). However, the pathway and the active ingredients of predominant *Lactobacilli* (*L. salivarius* and *L. agilis*) regulating ISCs require additional investigation.

Multiple studies illustrated that three primary bacterial metabolites, short-chain fatty acids (SCFAs), secondary bile acids (BAs), and tryptophan metabolites (indole-based metabolites), play crucial roles in the maintenance of gut epithelial integrity (19). What is more, SCFAs and indole-based metabolites (such as indole 3-carbinol), exert the effect of anti-proliferative and prodifferentiative on ISCs (20, 21). The indole-3-aldehyde, a L. reuteri D8 metabolite, was verified to stimulate lamina propria lymphocytes (LPLs) to secret IL-22 through aryl hydrocarbon receptor (AhR), then induced phosphorylation of STAT3 to accelerate the proliferation of intestinal epithelial (22). Additionally, the exopolysaccharides from *L. plantarum* NCU116 were demonstrated to modulate mice’s intestinal epithelial regeneration and promote the expansion of ISCs *in vivo* (23). Concerning *L. salivarius* or *L. agilis*, Xia et al. (24) pointed out that the colonization of *L. salivarius* LI01 in rats would produce indole-3-lactic acid and enhance arginine metabolism in the intestine, exerting an effect on energy accumulation in the liver. Shi et al. (25) suggested that *L. agilis* 32 was tolerant to the gastrointestinal environment of chicken and processed an excellent antibacterial effect on E. coli 10, primarily related to its metabolites or low pH conditions. Recently, intestinal microbes derived succinate was demonstrated to induce tuft cell expansion by increasing the TCA cycle (tricarboxylic acid cycle), thus further reducing inflammation in mice’s Crohn’s-like ileitis (26). Despite this, the active ingredient and the possible pathway that mediated the regulation of *Lactobacilli* on ISCs remain poorly understood.

In this study, the preliminary screening of active ingredient of predominant *Lactobacilli* was performed by component feeding trial. The metabolomics assay of *Lactobacillus* supernatant was further conducted to uncover the active ingredient that mediated the direct regulation of *Lactobacillus* on ISCs activity. Afterward, the active ingredient was administration to ISCs *in vivo* and *in vitro* to confirm its direct regulation and explore the underlying mechanism.

## RESULTS

### Effect of *Lactobacillus* component feeding on ISCs activity.

**FIG 1:**
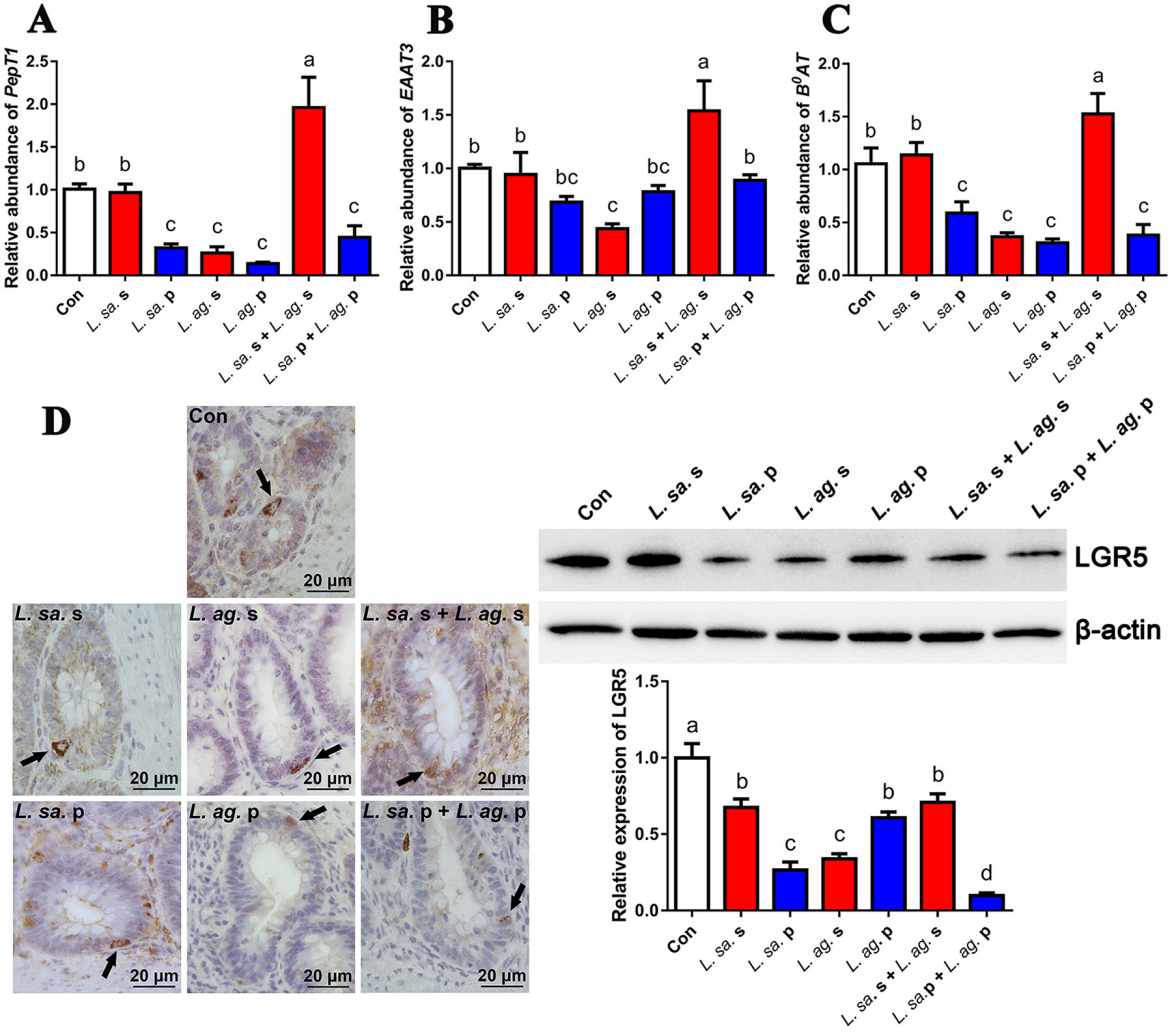
Effect of *Lactobacillus* component feeding on ISCs activity. The laying hens were fed with *Lactobacillus* supernatant or precipitation for 40 consecutive days. (A–C) The mRNA transcription assay of duodenal amino acid/peptide transporter (*PepT1*, *EAAT3*, *B^0^AT*) at the end of the feeding trial. (D) The assay of LGR5 protein, a marker of active ISCs, in the duodenal crypt after the feeding trial by immunohistochemistry staining and Western blot, black arrow indicates LGR5^+^ ISCs, scale bar = 20 μm. Con, control; *L. sa.* s, *L. salivarius* supernatant; *L. sa.* p, *L. salivarius* precipitation; *L. ag.* s, *L. agilis* supernatant; *L. ag.* p, *L. agilis* precipitation. The data were expressed as mean ± standard deviation (*n* = 3), and the columns with no common letters showed significant differences (*P* value < 0.05) among various treatments.

To evaluate the regulation of *Lactobacillus* components (supernatant, precipitated bacteria) on mucosal absorption, the mRNA abundance of amino acid/peptide transporters in the duodenum was analyzed at the end of feeding trial. As shown in Fig. 1A to C, the mRNA abundance of amino acid/peptide transporters were decreased at various degree upon *Lactobacillus* component feeding, except significantly increased in *L. salivarius* + *L. agilis* supernatants group (*PepT1*, increased by 94.22%, *P* value < 0.0001; *EAAT3*, increased by 53.58%, *P* value = 0.009; *B^0^AT*, increased by 44.94%, *P* value = 0.008). Furthermore, the expression of ISCs marker (LGR5) was analyzed. As shown in Fig. 1D, LGR5^+^ cells were located at the base of the intestinal crypts in a triangular wedge shape (black arrows), and didn’t show obvious morphological change upon *Lactobacillus* component feeding. However, the relative expression of LGR5 protein in the crypt decreased upon *Lactobacillus* component feeding. Among various groups, the repression of *L. salivarius* supernatant and *L. salivarius* + *L. agilis* supernatants on LGR5 expression was mild. And the LGR5 level in *L. salivarius* supernatant group was significantly higher by 99.8% (*P* value < 0.0001) than *L. agilis* supernatant group. Thus, we hypothesized that, due to the bacteriostatic activity of *Lactobacilli*, the stimulation from host intestinal flora to ISCs *in vivo* was alleviated. Simultaneously, some active ingredients existed in *L. salivarius* supernatant to promote ISCs activity.

### Host intestinal flora participated in the regulation of *Lactobacillus* feeding on ISCs activity.

**FIG 2:**
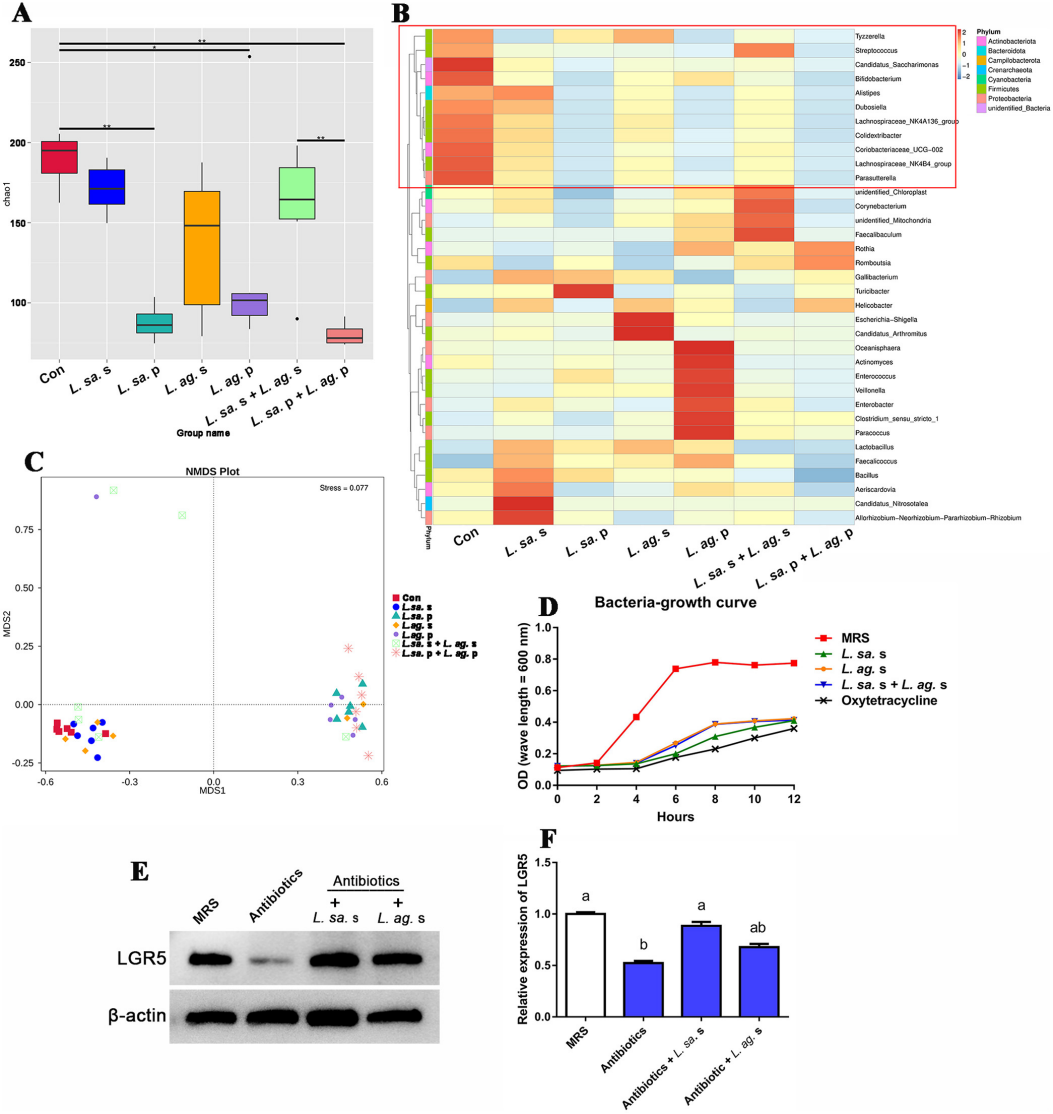
Effect of *Lactobacillus* component feeding on the host intestinal flora. After the *Lactobacillus* component (supernatant or precipitation) feeding trial, the intestinal contents from hens were collected and analyzed by 16S rRNA genes sequencing (*n* = 6). (A) Chao1 richness estimate. (B) The heatmap of microbial composition at genus-level of top 35. (C) Non-Metric Multi-Dimensional Scaling analysis plots (NMDS). In addition, the bacteriostatic ability of *Lactobacillus* supernatant was evaluated by the bacterial growth curve (D). (E–F) The Western blot assay of LGR5 protein after the feeding of antibiotics (Ampicillin Sodium, Neomycin Sulfate, Metronidazole, and Vancomycin HCl) in hens (*n* = 3). MRS, treated with MRS as control; *L. sa.* s, *L. salivarius* supernatant; *L. sa.* p, *L. salivarius* precipitation; *L. ag.* s, *L. agilis* supernatant; *L. ag.* p, *L. agilis* precipitation. The data were expressed as mean ± standard deviation, *, *P* value < 0.05; **, *P* value < 0.01, and the columns with no common letters showed significant differences (*P* value < 0.05) among various groups.

To evaluate whether the decreased ISCs activity resulted from the alleviated stimulation from gut microorganisms, the intestinal flora sequencing was performed. The results showed that chao1 index (Fig. 2A) in precipitated bacteria groups (*L. salivarius* p, *L. agilis* p, and *L. salivarius P+L. agilis* p) were all prominently lower than that in the control group. Meanwhile, the Non-Metric Multi-Dimensional Scaling analysis plots (NMDS, Fig. 2C) revealed an entirely different community composition between groups of precipitated bacteria and control. Furthermore, the heatmap (Fig. 2B) of microbial composition at the genus-level of top 35 illustrated that, versus the control group, feeding precipitated bacteria considerably reduced the abundance of the intestinal microbiota in 11 genera (indicated by the red box, which includes pathogenic bacterium such as streptococcus, etc.). Meanwhile, when incubating intestinal bacteria from healthy hens *in vitro*, the administration of *Lactobacillus* supernatant singly or combined was demonstrated to inhibit the viability of bacteria (Fig. 2D), which was even close to that of antibiotic (Oxytetracycline). These confirmed that *L. salivarius* and *L. agilis* exerted pronounced bacteriostatic effects through live bacteria and their supernatant.

To verify whether alleviating stimulation of gut microbiota would decrease the activity of ISCs, the chickens were fed with multiple antibiotics. The evaluation of ISCs activity (Fig. 2E and F) showed that upon antibiotics feeding, the protein level of LGR5 was dramatically reduced by 47.64% (*P* value = 0.002) versus the control group. This confirmed that reducing bacteria abundance in the gut lumen would mitigate the stimulation from gut microbiota to ISCs. What’s more, compared with the antibiotics feeding group, the administration of *L. salivarius* supernatant following antibiotics feeding was shown to elevated LGR5 expression by 69.00% (*P* value = 0.001), while *L. agilis* supernatant only elevated the LGR5 expression by 29.52% (*P* value = 0.092). These implied that some active ingredients existed in *L. salivarius* supernatant to promote the ISCs differentiation directly.

### Effect of *Lactobacillus* supernatant on ISCs activity *in vitro*.

**FIG 3:**
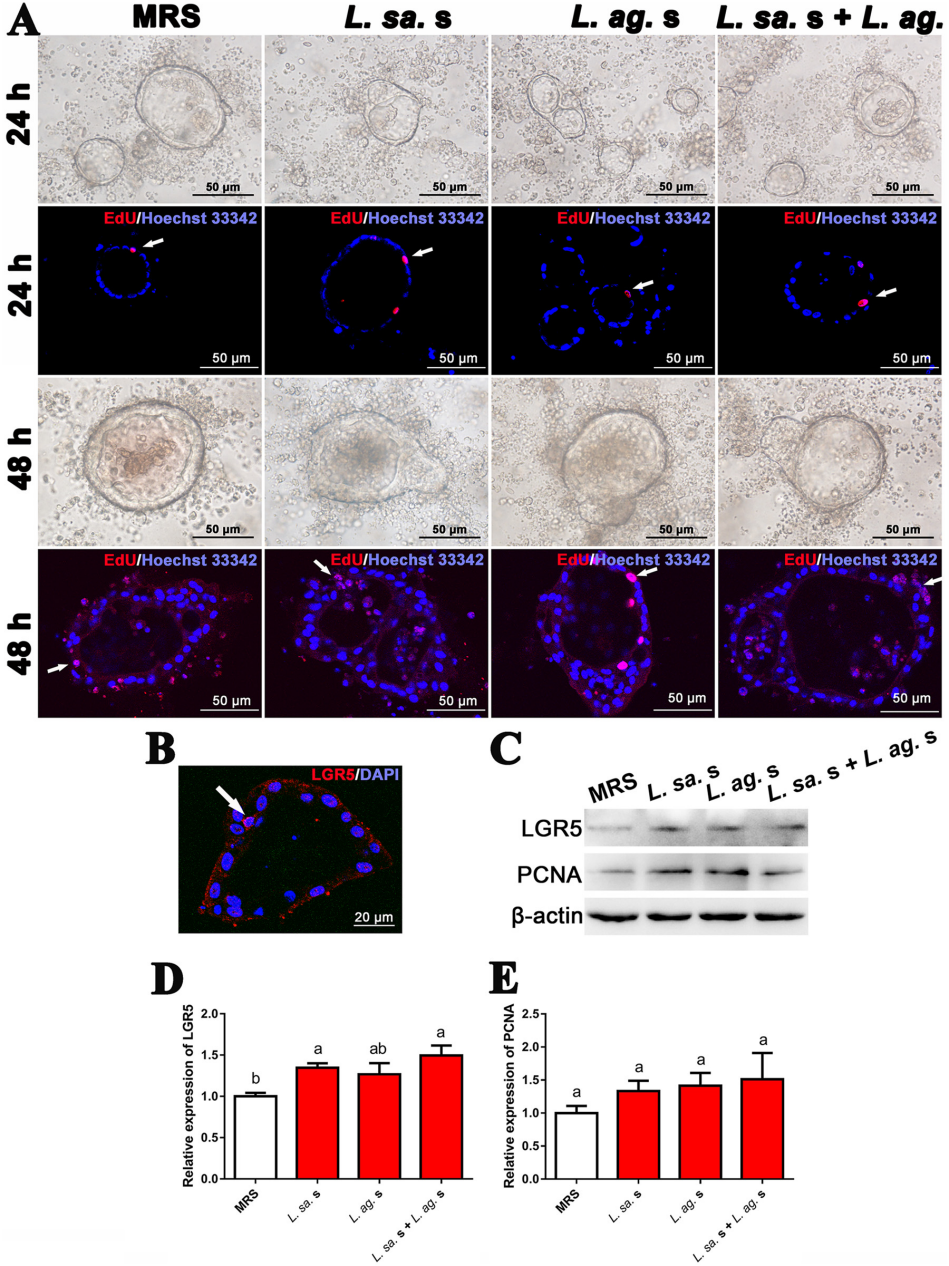
Effect of *Lactobacillus* supernatant on ISCs activity in the intestinal organoids. (A) In vitro, the intestinal organoids were cultured in a medium supplied with MRS (control) or *Lactobacillus* supernatant for 48 h. Meanwhile, the EdU incorporation assay was performed, white arrows indicate EdU^+^ cells, scale bar = 50 μm. (B) The immunofluorescent staining of LGR5 protein in the intestinal organoids, white arrow indicates LGR5^+^ ISC, scale bar = 20 μm. (C) Western blot assay of LGR5 and PCNA protein in the intestinal organoids. (D–E) The histograms represent the statistical results of relative expression of LGR5 and PCNA protein in the intestinal organoids. *L. sa.* s, *L. salivarius* supernatant; *L. ag.* s, *L. agilis* supernatant. The data were expressed as mean ± standard deviation (*n* = 3), and the columns with no common letters showed significant differences (*P* value < 0.05) among various treatments.

To exclude the disturbance of host intestinal flora and to clarify the direct regulation of *Lactobacillus* supernatant on ISCs, we established three-dimensional cultured intestinal organoids *in vitro* and cocultured them with specific *Lactobacillus* supernatant for 48 h. Compared with the control group (treated with MRS), larger organoids’ size and more budding structures appeared after treating *Lactobacillus* supernatant for 48 h (Fig. 3A). Furthermore, the analysis of LGR5 protein expression in intestinal organoids was performed. Similar to that *in vivo*, LGR5^+^ cells presented a triangular wedge shape (Fig. 3B, white arrow). The LGR5 protein level (Fig. 3C and D) in the group of *L. salivarius* supernatant and *L. salivarius* + *L. agilis* supernatants was remarkably upregulated by 34.51% (*P* value = 0.036) and 49.52% (*P* value = 0.007) compared with the control group. The proliferation activity assay showed that the EdU^+^ cells were located at the epithelium of organoids (Fig. 3A), and the relative expression of PCNA protein in *Lactobacillus* supernatant groups increased by 33.33 to 51.06% than the control group, however without statistical difference (Fig. 3C and E). These suggested that the supernatant of *L. salivarius* could promote the ISCs differentiation directly.

### Screening of active ingredients that mediated the promotion of *Lactobacillus* on ISCs activity.

**FIG 4:**
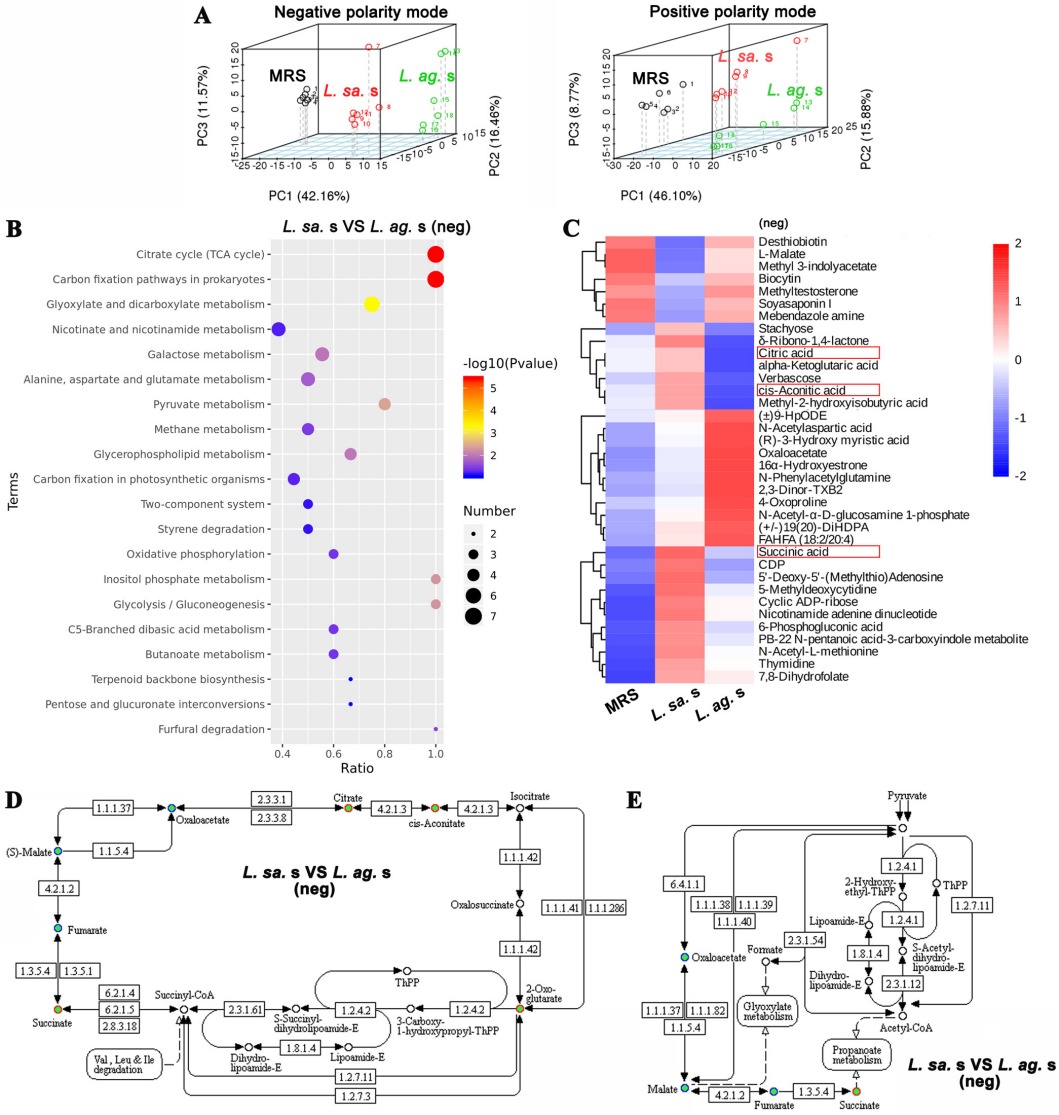
The untargeted metabolomics analysis of *Lactobacillus* supernatant. (A) Principal-component analysis (PCA) of *Lactobacillus* supernatant in negative and positive polarity mode. (B) Scatterplot of KEGG pathways enriched in negative polarity mode. (C) Hierarchical clustering analysis of metabolites related to TCA cycle (Citrate cycle). (D, E) KEGG enrichment pathway map of mitochondrial TCA cycle and microbial Pyruvate metabolism. *L. sa.* s, *L. salivarius* supernatant; *L. ag.* s, *L. agilis* supernatant.

To screen the active ingredients in *Lactobacillus* supernatant that facilitates intestinal organoids development, the supernatant of *L. salivarius* and *L. agilis* was subjected to an untargeted metabolomics analysis. Principal-component analysis (PCA, Fig. 4A) showed a significant separation in metabolites among *L. salivarius*, *L. agilis*, and MRS in both negative and positive polarity modes. Compared with the MRS, 176 differential metabolites were identified in the *L. salivarius* supernatant, and 205 differential metabolites were identified in the *L. agilis* supernatant. Next, we accessed the classification information of all identified metabolites through HMDB database annotation and found that the number of organic acids and derivatives-related metabolites was the highest in HMDB, both in negative and positive polarity mode.

The KEGG pathway (Kyoto Encyclopedia of Genes and Genomes) assay showed that, compared with *L. agilis* supernatant, differential metabolites in *L. salivarius* supernatant were noticeable enriched in the TCA cycle (Fig. 4B). The hierarchical clustering analysis further confirmed that the enriched metabolites in *L. salivarius* supernatant were related to the TCA cycle, such as succinate, citric acid, and cis-aconitic acid (Fig. 4C, red boxes). Interestingly, the increased succinate appeared both in the mitochondrial TCA cycle (Fig. 4D) and the microbial Pyruvate metabolism pathway (Fig. 4E). Based on the phenomenon that the promotion of *L. salivarius* supernatant on ISCs was stronger than *L. agilis* supernatant both *in vivo* and *in vitro*, we hypothesized that succinate might be a critical metabolite that exerts the promotion of *L. salivarius* on ISCs activity.

### Promotion of succinate on ISCs activity and the underlying mechanism.

**FIG 5:**
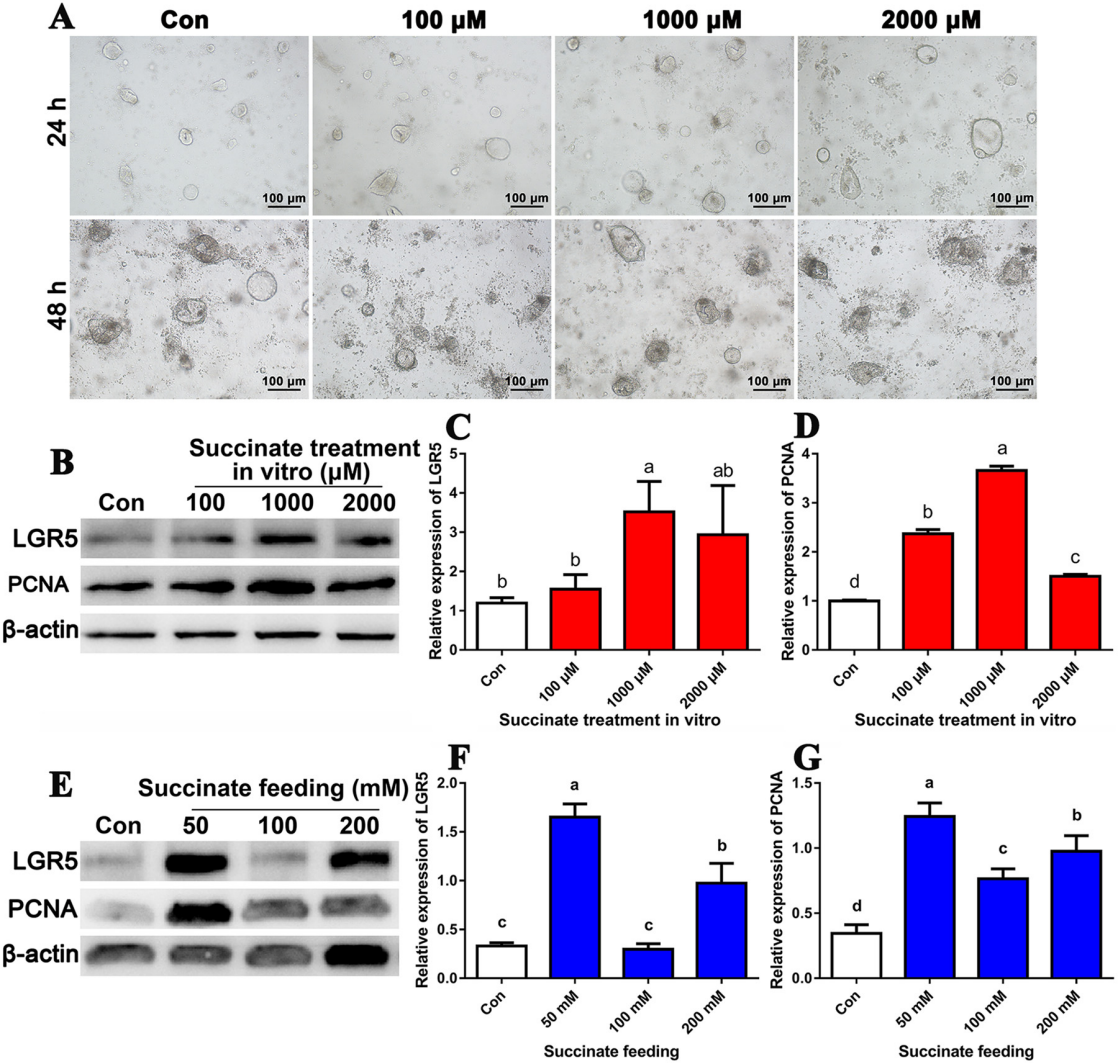
Effect of succinate on ISCs *in vitro* and *in vivo*. The intestinal organoids were treated with succinate (100 μM to 2000 μM) for 2 days, meanwhile, the hens were fed with succinate (50 mM to 200 mM) for 40 days. (A) The morphological alteration of intestinal organoids upon succinate treatment, scale bar = 100 μm. The Western blot assay and the histograms represent the alteration of LGR5 and PCNA protein levels upon succinate treatment *in vitro* (B–D) and *in vivo* (E–G). The data were expressed as mean ± standard deviation, and the columns with no common letters showed significant differences (*P* value < 0.05) among various treatments, *n* = 3.

To further characterize the effect of succinate on ISCs *in vitro* and *in vivo*, the intestinal organoids were treated with succinate (100 μM to 2000 μM); meanwhile, the laying hens were fed with succinate (50 mM to 200 mM). As shown in Fig. 5A, the intestinal organoids grew well upon succinate treatment. At 48 h of succinate treatment *in vitro*, the size and budding structures of intestinal organoids were augmented at the dosage of 100 μM and 1000 μM. The protein expression assay (Fig. 5B to G) showed that, compared with the control group, the 1,000 μM succinate (*in vitro*) and 50 mM succinate (*in vivo*) ascended expression of LGR5 protein (by 194.77% and 399.45%, *P* value = 0.008 and < 0.0001) and PCNA protein (by 266.00% and 259.79%, *P* value < 0.0001 and 0.001).

**FIG 6:**
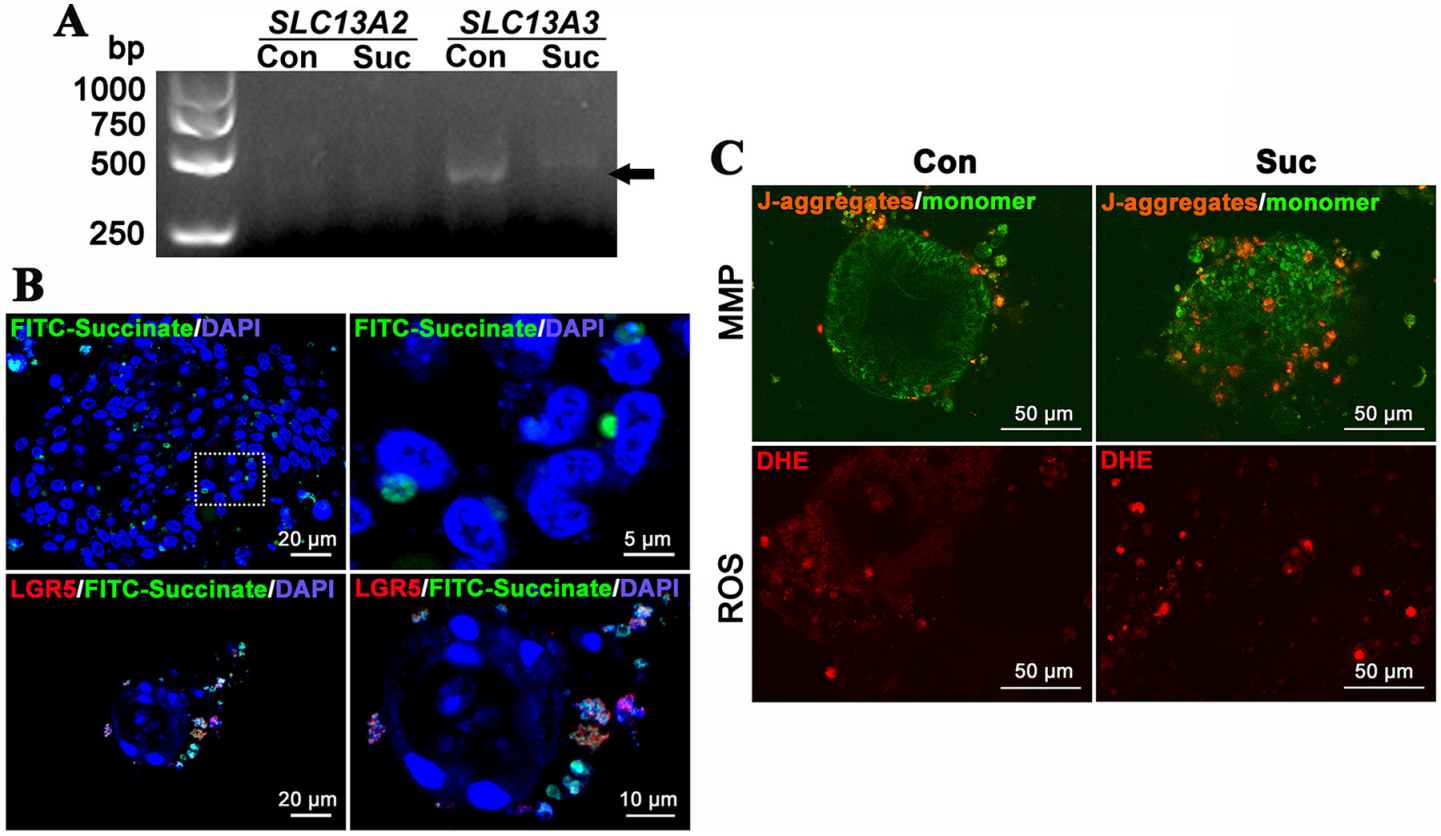
Succinate enhanced mitochondrial energy metabolism in ISCs. (A) RT-PCR assay of succinate transporter, NaDC-1 (*SLC13A2*) and NaDC-3 (*SLC13A3*), in the intestinal organoids. (B) Upon the treatment of FITC-succinate in intestinal organoids for 24 h, the FITC-succinate entered the cytoplasm and contacted the nucleus of ISCs. The right panel shows enlarged images of the left panel. (C) JC-1 and DHE staining were performed to evaluate the mitochondrial membrane potential (MMP) and reactive oxygen species (ROS) levels in intestinal organoids upon the succinate treatment, scale bar = 50 μm. Con, Control; Suc, Succinate.

To gain in-depth insight into the pathway that succinate acts on ISCs, it is a breakthrough to identify whether the succinate can enter ISCs. First, sodium/dicarboxylate cotransporters (*SLC13A2* and *SLC13A3*) were analyzed by RT-PCR. As presented in Fig. 6A (black arrow), *SLC13A3* existed in the intestinal organoids. Importantly, succinate could enter the cytoplasm of ISCs. As illustrated in Fig. 6B, after the treatment of FITC-succinate in intestinal organoids for 24 h, FITC-succinate appeared in the cytoplasm and contacted the nucleus of ISCs, as confirmed by the colocalization of FITC-succinate and LGR5 protein. Subsequently, the mitochondrial membrane potential (MMP) was analyzed by a JC-1 probe. The results revealed that orange J-aggregates were enhanced obviously in the succinate group (Fig. 6C), indicating the elevated mitochondrial activity induced by succinate. As a by-product of OXPHOS, ROS was further analyzed by DHE staining. As shown in Fig. 6C, ROS level was increased after succinate treatment, suggesting an enhanced energy metabolism in the intestinal organoids.

## DISCUSSION

In our previous study, cofeeding of *L. salivarius* and *L. agilis* in laying hens was demonstrated to decrease the ISCs differentiation (11). Currently, after the administration of *Lactobacillus* component (supernatant, precipitation) on hens individually or combined, the LGR5 protein expression *in vivo* was downregulated, the mRNA abundance of amino acid/peptide transporters presented a similar alteration trend. It is well known that the niche of most stem cell populations maintains stem cell quiescence and prevents differentiation (27). In contrast, bacterial cell wall-derived antigens would drive the differentiation of ISCs. For instance, Gram-positive commensal bacteria were a major determinant of enterocyte turnover in mice (28). Liu et al. (29) suggested microbiota promote ISCs differentiation into enterocytes. Recently, a key pathophysiology event in inflammatory bowel disease (IBD) has been established as rewiring the ISCs niche by pathogenic or commensal microbiota (20). In our study, the strong bacteriostatic activity of *L. salivarius* supernatant and *L. agilis* supernatant were verified. In agreement, *L. salivarius* and *L. agilis* 32 were previously proved to present an excellent antibacterial effect on pathogenic bacterium of humans or chickens (25, 30). These implied that the stimulation from gut flora to ISCs was alleviated after feeding *L. salivarius* or *L. agilis*. Consistently, after multiple antibiotics feeding, the protein expression of LGR5 was also remarkably decreased. We thus proposed that *L. salivarius* and *L. agilis* feeding could maintain ISCs quiescence and prevent differentiation *in vivo* due to their bacteriostatic activity.

Although *Lactobacillus* feeding decreased the expression of LGR5, the repression of *L. salivarius* supernatant and *L. salivarius* + *L. agilis* supernatants on ISCs were mild. These indicated that, apart from bacteriostatic activity, some active ingredients existed in the *L. salivarius* supernatant to promote ISCs activity. Further, the administration of *L. salivarius* supernatant following antibiotics feeding was shown to promote ISCs differentiation when compared with the antibiotics feeding group, which confirmed this speculation. In line with our expectation, the treatment of supernatant (*L. salivarius* and *L. salivarius + L. agilis*) *in vitro*, excluding the disturbance of host microbiota, remarkably enhanced the LGR5 protein expression in intestinal organoids. These confirmed our speculation that, in *L. salivarius* supernatant, active ingredients indeed existed to accelerate ISCs activity. Similarly, L. reuteri was demonstrated to enhance the expression of LGR5 protein and promote proliferation activity in the intestinal organoids of mice (31). Next, the metabolomics analysis of the *Lactobacillus* supernatant showed that, between *L. salivarius* and *L. agilis*, differential metabolites of *L. salivarius* supernatant were noticeably enriched in the TCA cycle. Among them, succinate caused our concern, for it participates in both the mitochondrial TCA cycle and microbial Pyruvate metabolism pathway (Fig. 4D and E). It has long been recognized that the luminal succinate is derived from commensal microbiota rather than the host (26). That means succinate bridges the gap between microbial and cellular energy metabolism of the host. Previously, 5,000 μM succinate was used to enhance intestinal epithelial barrier function in pigs (32), and 50 μM succinate was used to accelerate hMSC migration (33). In the present study, after the treatment of succinate on ISCs *in vitro* and *in vivo*, it appeared that the 1,000 μM (*in vitro*) and 50 mM (*in vivo*) succinate could significantly enhance the expression of LGR5 and PCNA. We thus proposed that succinate, a metabolite in *L. salivarius* supernatant, could promote the ISCs activity.

In the case of the mechanism that succinate acting on ISCs, Connors et al. (34) reported that within the gastrointestinal tract of humans, sodium/dicarboxylate cotransporter 1 (NaDC-1, SLC13A2) is the predominant succinate transporter and expressed on the apical face of enterocyte. Besides, human renal NaDC-3 (SLC13A3) could also transport four carbon dicarboxylates, including succinate (35). In the present study, *SLC13A3* was demonstrated to exist in the intestinal organoids. It could transport succinate into ISCs, as illustrated by the colocalization of FITC-succinate and LGR5 in the intestinal organoids. Previously, intracellular succinate was demonstrated to promote the differentiation of mice’s embryonic stem cells *in vitro* (36). In addition, succinate was shown to promote the TCA cycle gene in tuft cells and further trigger the tuft cell-ILC2 circuit that drives crypt niche alteration (26, 37), Moreover, succinate-induced intracellular mitochondrial ATP enhancement was verified to promote hMSC migration (33). In the present study, the elevated MMP and ROS levels in the intestinal organoids further confirmed the enhanced mitochondrial OXPHOS. Taken together, it is reasonable to presume that the succinate entering ISCs would enhance mitochondria function and thus promote the differentiation of ISCs.

**FIG 7:**
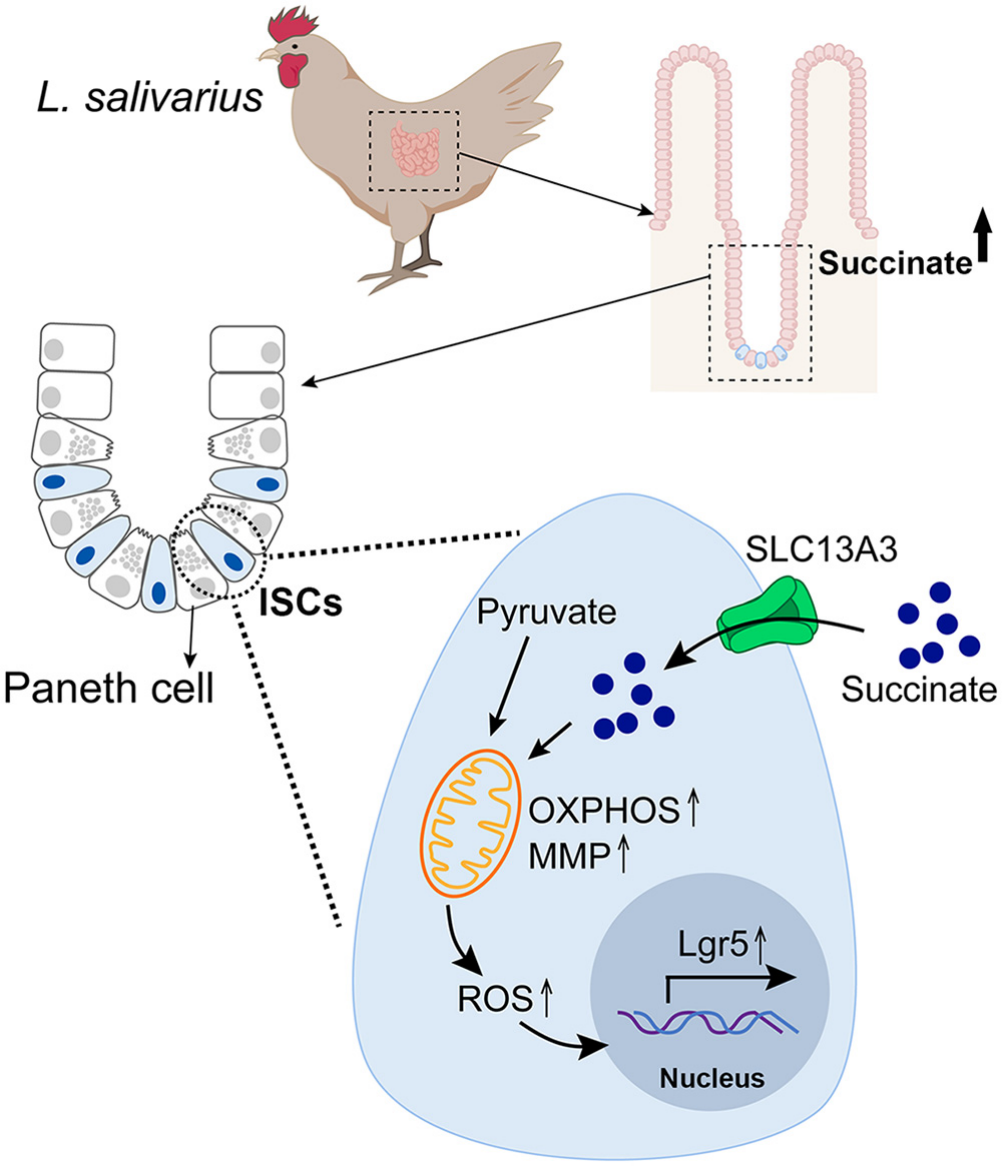
Schematic representations of the main findings in this work. Upon *L. salivarius* feeding, the succinate in supernatant could be transported into ISCs through SLC13A3 (a sodium/dicarboxylate cotransporter), subsequently promoting the ISCs activity by elevating mitochondrial membrane potential (MMP) and reactive oxygen species (ROS) levels.

To summarize our findings, as illustrated in Fig. 7, succinate is one metabolite in the *L. salivarius* supernatant that can be transported into ISCs and further promotes the ISCs activity by elevating MMP and ROS levels.

## MATERIALS AND METHODS

### *Lactobacilli* and their component preparation.

*L. salivarius* (CGMCC 1.1881) and *L. agilis* (CGMCC 1.3914) were purchased from China General Microbiological Culture Collection Center (CGMCC) and cultured in MRS agar medium (Beijing Land Bridge Technology Co., CM187, China) at 37°C for 48 h. Under the aseptic condition, single colonies of the two strains of *Lactobacillus* were picked into MRS liquid medium and further incubated at 37°C for 24 h, followed by adjusting the concentration of the bacterial solution by MRS to 10^8^ CFU/mL and OD_660_ = 0.400. The bacterial supernatant and precipitation of these *Lactobacilli* were obtained by centrifuging at 1250 × *g* for 2 min under 4°C. The supernatant was further bacteriological filtration by using a 0.22-μm syringe filter.

### Animal feeding trial and experimental design.

A total of 210 HyLine (HL) hens at the age of 280-day-old (D280, laying peak period) were raised on a large-scale hen farm (Shengxin laying hens breeding cooperative, Nanping, Fujian, China), fed with the basal diet, and provided free access to water. All birds were randomly divided into seven groups (*n* = 30, 10 hens/cell × three cells/group). One control group (fed the basal diet) and six *Lactobacillus* component supplement groups include (i) *L. salivarius* supernatant group, (ii) *L. salivarius* precipitation group, (iii) *L. agilis* supernatant group, (iv) *L. agilis* precipitation group, (v) *L. salivarius* supernatant + *L. agilis* supernatant group, and (vi) *L. salivarius* precipitation + *L. agilis* precipitation group, respectively, fed with a basal diet containing corresponding *Lactobacillus* component for 40 consecutive days. On the 40^th^ day of the trial, six hens in each group were randomly collected for sampling.

To prove intestinal flora regulation on mucosal structure, 20 HL hens were divided into four groups (*n* = 5) include (i) MRS group (fed the MRS as control), (ii) antibiotics feeding group, (iii) antibiotics + *L. salivarius* supernatant group, and (iv) antibiotics + *L. agilis* supernatant group. In groups ii to iv, hens were supplied with water containing Ampicillin Sodium (1 g/L water, MB1378, Meilunbio, Dalian, China), Neomycin Sulfate (1 g/L water, MB1716, Meilunbio, Dalian, China), Metronidazole (1 g/L water, MB2200, Meilunbio, Dalian, China), and Vancomycin HCl (0.5 g/L water, MB1260, Meilunbio, Dalian, China) for 2 weeks. On the second week of antibiotics feeding, *L. salivarius* supernatant or *L. agilis* supernatant was added to groups iii and iv. On the 14^th^ day of the trial, five hens in each group were collected for sampling.

For analyzing the effect of succinate on ISCs *in vivo*, a total of 20 HL hens (D280) were fed with succinate (Mb4700, Meilunbio, Liaoning, China) for 40 days. All birds were randomly divided into four groups (*n* = 5): (i) control group, (ii) 50 mM succinate group, (iii) 100 mM succinate group, and (iv) 200 mM succinate group, which were fed with the basal diet and free access to water or water containing succinate at a specific concentration. On the 40^th^ day of the trial, five hens in each group were collected for sampling.

This study was carried out following the Guiding Principles for the Care and Use of Laboratory Animals of Zhejiang University. The experimental protocols were approved by the Committee on the Ethics of Animal Experiments of Zhejiang University (No. 14933).

### Crypt isolation and intestinal organoids culture.

Isolation of chicken’s intestinal crypt was performed based on the protocol previously published (11, 12). Briefly, the duodenum was collected from HL hens at D280 (laying peak period) sterility, cut into 2–4 mm^3^ pieces and shaken gently in 2 mM cold EDTA (ethylenediaminetetraacetic acid, pH 7.4). After passing through a 70-μm nylon cell strainer (Corning Inc., 352360; Corning, NY, USA), the crypts were purified by centrifugation (100 × *g*) and resuspended. The purified crypt was further used for transcriptomics analysis, protein expression assay, and intestinal organoids culture.

For intestinal organoids culture, the sterilely purified crypts were mixed with Matrigel (354277, Corning), dropped into a flat-bottom cell culture plate, and administered with a complete culture medium (the advanced DMEM/F12 culture medium comprising 50 ng mL^−1^ EGF, 100 ng mL^−1^ Noggin and 500 ng mL^−1^ R-spondin 1), then cultured at 37°C in a 5% CO_2_ atmosphere and the crypts will budding to form organoids. At the beginning of the culture, the 250 μL of bacterial supernatant (*L. salivarius* or *L. agilis* with 10^4^ CFU/mL) or succinate (100 μM-2000 μM, Mb4700, Meilunbio, Liaoning, China) or FITC-succinate (1,000 μM, Xi’an Ruixi Biological Technology Co. Ltd., customization, Shaanxi, China) were administered into the medium. At 24 h and 48 h of culture, the intestinal organoids were collected for the assay of morphology, mRNA/protein expression, and mitochondrial activity.

To analyze the proliferation activity, the intestinal organoids were incorporated with EdU (C0078S, Beyotime, Shanghai, China) for 2 h, followed by fixed in 4% PFA solution for 20 min and incubated with click-it reaction solution for 30 min. To analyze the mitochondrial activity, the intestinal organoids were incubated with a JC-1 probe (C2006, Beyotime, Shanghai, China) for 30 min at 37°C, the JC-1 dye accumulated in mitochondria as a monomer (green fluorescence) at low mitochondrial membrane potential (MMP), while forms J-aggregates (red/orange fluorescence) at high MMP. To analyze the reactive oxygen species (ROS) levels, the intestinal organoids were incubated with the hydroethidine (DHE) reagent (D23107, Thermo Fisher Scientific, Waltham, MA, USA), which dissolved in PBS with a final concentration of 10 μM, for 30 min at 37°C. To analyze the location of LGR5^+^ ISCs, the intestinal organoids were incubated with rabbit-anti chicken LGR5 antibody (1:50, HuaBio, customization, Hangzhou, China) at 4°C overnight, followed by a routine immunofluorescent staining (IF) protocol. The nuclei were counterstained by Hoechst 33342 for 10 min or 4′,6-diamidino-2-phenylindole (DAPI) for 20 min, and the images were taken by laser scanning confocal microscope (Olympus IX81-FV1000, Japan).

### Protein expression and mRNA transcription assay.

For assaying the expression of a specific protein, the Western blot (WB) was performed. Briefly, the total protein (extracted from the duodenal crypt and intestinal organoids) was transferred to a polyvinylidene fluoride membrane (0.22 μm) following SDS-PAGE. After rinsing and blocking, the membrane was incubated with primary antibodies (LGR5, 1:800, HuaBio, customization, Hangzhou, China; PCNA, 1:500, Abcam, ab29, Cambridge, UK; β-actin, 1:25000; Abclonal, AC026, Wuhan, China) overnight at 4°C. After rinsing, the membrane was incubated with HRP-conjugated secondary antibody (1:10,000, HuaBio, Hangzhou, China) for 1 h at 37°C. For the semiquantitative assay, images were analyzed using the Gel-Pro/Image J software, and the gray analysis of target protein bands was normalized with β-actin.

The quantification Real-Time PCR (qPCR) or RT-PCR were performed base on the protocol previously published (11). Briefly, total RNA from the intestinal tissue or organoids were extracted using TRIzol reagent and reverse transcribed into cDNA. The cDNA was used as the template amplification. The primers used were listed as follows: *PepT1* (NM_204365, F: GATCACTGTTGGCATGTTCCT; R: CATTCGCATTGCTATCACCTA); *EAAT3* (XM_424930, F: AAAATGGGAGACAAAGGACAA; R: ACGAAAGATTTCCCAGTCCTC); *B^0^AT* (XM_419056, F: AATGGGACAACAAGGCTCAG; R: CAAGATGAAGCAGGGGGATA); *SLC13A2* (XM_011525452.2, F: GCCATCAGCATCCTATTCGTCATCC; R: GCATCTTCTGGTTCACCGTCTTCC); *SLC13A3* (XM_040688600.2, F: AACACGGCGACCATCATCATCTTC; R: TGTGCCCAGGTATTCATAGCCAAAC); *GAPDH* (NM_204305.1, F: GATGGGTGTCAACCATGAGAAA; R: CAATGCCAAAGTTGTCATGGA). All samples were measured in triplicate, and the experiments were repeated more than three times. All samples were normalized with *GAPDH* using the comparative cycle threshold method (2^−ΔΔCt^).

### Bacteriostatic curve.

The small intestinal contents of healthy hens were collected with sterile PBS. First, the supernatant of the contents was inoculated into a BHI medium and incubated at 37°C for 24 h. Next, single colonies were picked in BHI liquid medium and incubated at 37°C for 24 h to obtain bacterial solution. After that, 1 mL bacterial broth was added to a 9 mL *Lactobacillus* supernatant and incubated for 12 h at 37°C. Simultaneously, 9 mL MRS broth medium and Oxytetracycline (10 mg/mL) were used for negative and positive control. During this period, the OD was detected at 600 nm wavelength every 2 h.

### Transcriptomics analysis.

For screening the differentially expressed genes in the crypt, the duodenal crypt was isolated from three hens in each group, frozen in dry ice, and mailed to a commercial company (Novogene Co. Ltd., Beijing, China). Briefly, total RNA was extracted, and the transcriptome sequencing was performed based on the Illumina sequencing platform. Significant differences in gene expression were detected using the EdgeR algorithm implemented in the CLC Genomics Workbench (Qiagen, Hilden, Germany). The *P* value threshold was determined by the false discovery rate (FDR) to account for multiple tests of significance. An FDR threshold ≤ 0.01 was adopted to judge the significance of the gene expression change throughout the tripartite bioassay.

### Intestinal flora sequencing.

For evaluating the alteration of intestinal microbial communities after feeding of *Lactobacillus* component in hens, the small intestinal contents from six hens in each group were collected sterilely, frozen in dry ice, and mailed to a commercial company (Novogene Co. Ltd., Beijing, China) for 16S rRNA genes V3-V4 region sequencing. Briefly, genomic DNA was extracted from intestinal flora according to the protocol, followed by PCR amplified using bar-coded primers flanking the V3-V4 region of the 16S rRNA genes. The extracted and purified amplicons were then pooled in equimolar and paired-end sequenced (2 × 250) on an Illumina MiSeq platform. Raw fastq files were demultiplexed and quality-filtered using QIIME (http://qiime.org/). Operational taxonomy units (OTUs) were clustered with 97% similarity cutoff using UPARSE (version 7.1 http://drive5.com/uparse/), and chimeric sequences were identified and removed using UCHIME. The taxonomy of each 16S rRNA genes sequence was analyzed by the RD *P* value < Classifier (http://rdp.cme.msu.edu/) against the silva (SSU115) 16S rRNA genes database using the confidence threshold of 70%.

### Untargeted metabolomics analysis.

To identify the metabolite of *L. salivarius* and *L. agilis*, the MRS, *L. salivarius* supernatant, and *L. agilis* supernatant were collected sterilely, frozen in dry ice, and mailed to a commercial company (Novogene Co. Ltd., Beijing, China) for untargeted metabolomics sequencing. Briefly, the samples were freeze-dried and re-suspend with prechilled 80% methanol by a vortex. Then the samples were incubated on ice for 5 min and centrifuged at 15,000 × *g*, 4°C for 15 min, and LC-MS/MS analysis were performed using a Vanquish ultrahigh-performance liquid chromatography system (UHPLC, Thermo Fisher Scientific) coupled with an Orbitrap Q Exactive HF-X mass spectrometer (Thermo Fisher Scientific) in both positive and negative modes. The raw data files generated by UHPLC-MS/MS were processed using the Compound Discoverer 3.1 (CD3.1, ThermoFisher) to perform peak alignment, peak picking, and quantitation for each metabolite. Afterward, peak intensities were normalized to the total spectral intensity. The normalized data were used to predict the molecular formula based on additive ions, molecular ion peaks, and fragment ions. The online KEGG database (https://www.genome.jp/kegg/pathway.html) was used to identify metabolites by matching the molecular mass data. The metabolites with variable-importance projection (VIP)-value > 1 and VIP-value < 0.05 and fold change (FC)-value ≥ 2 or FC-value ≤ 0.5 were considered differential metabolites.

### Statistical analysis.

Statistical analysis were performed using SPSS 16.0. For normally distributed data, one-way analysis of variance (ANOVA, followed by the Least Significant Difference *post hoc* multiple-comparison test) or independent *t* test was carried out. The significance level was set at *P* value < 0.05.

### Data availability.

The authors confirm that the data supporting the findings of this study are available within the article and its supplemental materials. The data sets supporting the conclusions of this article are available in the NCBI BioProject no. PRJNA847902 and PRJNA735554.
